# Do Women have More Barriers for Professional Development?

**DOI:** 10.1192/j.eurpsy.2022.199

**Published:** 2022-09-01

**Authors:** O. Kilic

**Affiliations:** Bezmialem Vakif University Faculty of Medicine, Department Of Psychiatry, Istanbul, Turkey

## Abstract

Women consistently fare worse than men in different professional metrics in terms of salary, promotions, grant money, and scholarly publishing. In clinical and management leadership roles, a similar trend of underrepresentation for women exists. It has been shown in academic publishing that women progressed to senior author positions at a slower pace, resulting in their ongoing underrepresentation as senior writers. Previous literature has shown that one of the most effective strategies for promoting women to leadership positions is the collaboration with men to identify attitudinal and institutional barriers, since gender equality is not only a “women’s concern.” To address this, a cross-sectional, multinational survey was disseminated in English (SurveyMonkey) to psychiatric trainees and psychiatrists across Europe. This study aims to explore the perspectives of female and male psychiatrists on the obstacles they face in their careers. The survey covered items on self-promotion, current institutional environment, and networking along with contextual information- age, gender, professional degree, current field, and years of professional experience in the current field. We hope that the presentation of these findings will serve as a springboard for future educational activities to address concerns connected to the gender gap. The speaker will present the comparison of female and male psychiatrists’ barriers and challenges they face during their professional development. This talk is hoped to elicit discussion in preparation for future action and inform a roadmap for addressing issues related to the gender gap with subsequent educational events.

**Disclosure:**

No significant relationships.

